# Effects of self-care, self-efficacy, social support on glycemic control in adults with type 2 diabetes

**DOI:** 10.1186/1471-2296-14-66

**Published:** 2013-05-24

**Authors:** Junling Gao, Jingli Wang, Pinpin Zheng, Regine Haardörfer, Michelle C Kegler, Yaocheng Zhu, Hua Fu

**Affiliations:** 1 School of Public Health, Fudan University, Key Laboratory of Public Health Safety, Ministry of Education, PO Box 248 138 Yixueyuan Road, Shanghai 200032, People’s Republic of China; 2 Dachang Center of Primary Health Care, Shanghai, People’s Republic of China; 3 Emory Prevention Research Center, Rollins School of Public Health, Emory University, Atlanta, GA 30322, USA

**Keywords:** Self-efficacy, Social support, Patient-provider communication, Glycemic control

## Abstract

**Background:**

A number of studies have examined the influence of self-efficacy, social support and patient-provider communication (PPC) on self-care and glycemic control. Relatively few studies have tested the pathways through which these constructs operate to improve glycemic control, however. We used structural equation modeling to examine a conceptual model that hypothesizes how self-efficacy, social support and patient-provider communication influence glycemic control through self-care behaviors in Chinese adults with type 2 diabetes.

**Methods:**

We conducted a cross-sectional study of 222 Chinese adults with type 2 diabetes in one primary care center. We collected information on demographics, self-efficacy, social support, patient-provider communication (PPC) and diabetes self-care. Hemoglobin A1c (HbA1c) values were also obtained. Measured variable path analyses were used to determine the predicted pathways linking self-efficacy, social support and PPC to diabetes self-care and glycemic control.

**Results:**

Diabetes self-care had a direct effect on glycemic control (β = −0.21, p = .007), No direct effect was observed for self-efficacy, social support or PPC on glycemic control. There were significant positive direct paths from self-efficacy (β = 0.32, p < .001), social support (β = 0.17, p = .009) and PPC (β = 0.14, p = .029) to diabetes self-care. All of them had an indirect effect on HbA1c (β =–0.06, β =–0.04, β =–0.03 respectively). Additionally, PPC was positively associated with social support (γ = 0.32, p < .001).

**Conclusions:**

Having better provider-patient communication, having social support, and having higher self-efficacy was associated with performing diabetes self-care behaviors; and these behaviors were directly linked to glycemic control. So longitudinal studies are needed to explore the effect of self-efficacy, social support and PPC on changes in diabetes self-care behaviors and glycemic control.

## Background

The prevalence of diabetes is high in China and continues to increase. Overall, 92.4 million Chinese adults 20 years of age or older (9.7% of the adult population) have diabetes, and in 60.7% of these cases, the diabetes is undiagnosed [1]. The main goals of diabetes management are to prevent microvascular and macrovascular complications [2] and to decrease mortality and economic costs due to diabetes. To achieve these goals, adequate glycemic control, including fasting blood glucose and glycosylated hemoglobin (HbA1c) has been recommended [3,4]. More and more studies indicate that self-care behaviors influence glycemic control [5-7]. But few patients engage in the full set of self-care behaviors at recommended levels [8,9]. Only 33% of Chinese patients performed daily foot care, and only 13% of patients performed blood glucose self-testing daily [10]. So it is important that diabetes care providers should understand factors influencing self-care behaviors.

One of the key factors in attaining active self-care is self-efficacy, a construct from social cognitive theory that focuses on one’s confidence to perform a given behavior [11]. Several studies have documented associations between self-efficacy and diabetes self-care [12-14]. Social cognitive theory also emphasizes the interplay between individual and environmental factors in shaping behavior. Previous studies [15-17] have demonstrated that social support, an environmental factor, has been shown to affect self-care and glycemic control. As a result, the American Dietetic Association (ADA) suggests that most patients need ongoing diabetes self-management support (DSMS) in order to sustain self-management behaviors at the levels needed to effectively manage diabetes [18]. Furthermore, many patients receive DSMS through their providers. Thus, communication is essential to ensure that patients receive the support they need. Studies indicate that good patient-provider communication (PPC) predicts better diabetes self-care, better diabetes outcomes, or both [19].

A number of studies have examined whether self-efficacy, social support and PPC are associated with glycemic control, but relatively few was conducted in China [10]. Although several studies have used structural equation modeling to examine similar psychosocial factors and diabetes management [10,20,21], few studies have examined these variables in one conceptual model. Understanding the pathways through which these variables interact to influence self-care behavior and glycemic control among Chinese patients with type 2 diabetes will aid in developing more effective interventions for this growing population.

**Figure 1:**
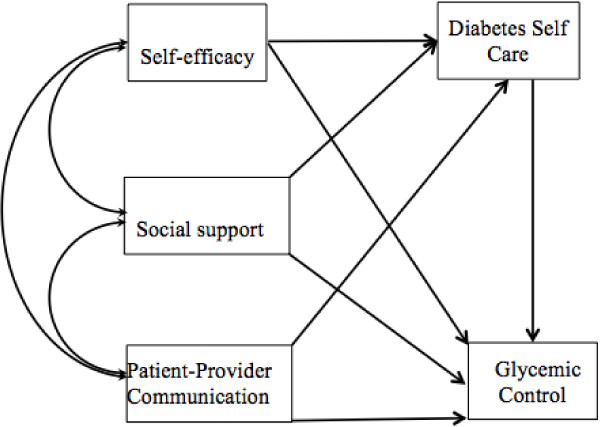
Hypothesized model of the relationship of self-efficacy, social support, patient-provider communication, and their effect on diabetes self-care and glycemic control.

The purpose of the current study is to explore the relationships of self-efficacy, social support and PPC, and their effects on self-care behaviors and glycemic control among Chinese patients with type 2 diabetes in a whole model. Based on literature review, we hypothesized that self-efficacy, social support and PPC would directly affect self-care behaviors and glycemic control; self-efficacy, social support and PPC would affect glycemic control via self-care behaviors; and social support, self-efficacy and PPC were associated with each other in Chinese patients with type 2 diabetes (Figure 1).

## Methods

A cross-sectional study was conducted at a primary health care center in Shanghai, China between June and October 2011. Participants were eligible if they received their usual care at the primary health care center and had a diagnosis of type 2 diabetes. We excluded participants if they could not complete the survey because of physical or cognitive impairments. All participants were provided written informed consent. The study was approved by the Institutional Review Board of the School of Public Health, Fudan University.

### Data and procedure

Research assistants reviewed the electronic clinic roster to identify eligible participants. Two hundred twenty-two participants of 274 eligible participants (response rate = 81%) were consented and completed the study. The consented eligible participants were invited to the center to complete the study survey, a physical examination and fasting blood glucose tests. Blood tests were provided for free as an incentive for participation. We collected data on self-reported age, gender, marital status, education, household income, family history of diabetes, and time diagnosed with diabetes. Research assistants checked all self-reported questionnaires for completeness.

Participants’ height, weight, waistline and hipline were measured as part of the physical examination. Body mass index(BMI) ≥ 24 is defined as overweight/obesity, Waist-to-hip ratio (WHR) > 0.90 for men, and >0.85 for women is defined as central obesity suggested by Chinese guideline on diabetes care [4].

Additional measures included validated surveys of self-efficacy, social support, PPC, and diabetes self-care behavior. Self-efficacy was assessed with the Chinese version of the Diabetes Management Self-Efficacy Scale (C-DMSES) containing 20 items [22]. It assesses the extent to which participants are confident they can manage nutrition, blood sugar monitoring, foot exams, physical exercise and weight, and medical treatment. Participants rated themselves on an 11 point scale ranging from “0 = can’t do at all” to “10 = certain can do”. The mean scores of the 20 items ranging from 0 to 10 were used to assess participants’ self-efficacy.

Self-care behavior was assessed with the 11-item revised Summary of Diabetes Self-Care Activities (SDSCA) scale [23]. Previous studies [22,24] indicated the revised SDSCA was suitable to measure self-care behavior of Chinese diabetes (Cronbach’s alpha = .70). The RSDSCA measures frequency of self-care activity in the last 7 days for six aspects of the diabetes regimen: general diet (followed healthful diet), specific diet (ate fruits/low fat diet), foot care, blood–glucose testing, taking medication and exercise. The Cronbach alpha coefficient of the Chinese version of the revised SDSCA in this study was 0.82, indicating good internal consistency.

Social support and PPC were measured using the Chinese versions of the questionnaires [25] based on The Health Education Impact Questionnaire [26]. Each scale consists of 5 items with a scoring range from 0 to 6 where 0 = “strongly disagree” and 6 = “strongly agree”. High scores of social support indicate high levels of social interaction, high sense of support, seeking support from others. The Cronbach alpha coefficient of social support scale was 0.930. High scores of PPC characterize a person who is confident in their ability to communicate with healthcare professionals and has good understanding of ways to access healthcare in order to get their needs met. The Cronbach alpha coefficient of PPC was 0.92.

Glycosylated hemoglobin (HbA1c) obtained on the same day as the survey served as the measure of glycemic control.

### Data analysis

Descriptive statistics, *χ*^2^ tests (for categorical variables) and ANOVAs (for continuous variables) to compare participants’ self-efficacy, social support, PPC, SDSCA, and HbA1c by demographics and key diabetic characteristics were performed using SPSS 17.0. Measured variable path analysis (MVPA), a form of structural equation modeling, was used to test the relationships among self-efficacy, social support and PPC, and their effect on self-care and glycemic control using AMOS 17.0. Simulation research has shown that with a good model and multivariate normal data a reasonable sample size is 200 cases [27]. The parameter estimation method was maximum likelihood. The likelihood ratio *χ*^2^ tests are reported, but model fit was primarily evaluated with the comparative fit index (CFI), standardized root mean residual(SRMR) and root mean square error of approximation (RMSEA) [28]. All of them test how well an estimated model fits the data structure. A non-significant likelihood ratio *χ*^2^ test suggests that the data fit the model well, while CFI values exceeding 0.90, SRMR and RMSEA values less than 0.08 indicate adequate model fit [29]. Variables included in the path model were normally distributed.

## Results

### Demographic characteristics of participants

Overall, 137 participants were female (61.7%), and most were married (92.3%). Participants were, on average, 54.5 years old (SD = 6.4, range: 44–80), but most were more than 60 years old (78.4%). Fifty-two graduated from technical school or college (23.4%). The prevalence of good glycemic control (HbA1c < 6.5%) was 52.7%. The mean value of HbA1c was 6.6% (SD = 1.2, range: 4.5–12.9). There were no significant differences among participants in assessments of their self-efficacy, social support, PPC, SDSCA and HbA1c based on demographic differences (see Table 1).

**Table 1:** Demographic characteristics of participants

**Characteristic**	**N(%)**	**Self-efficacy**		**Social support**		**PPC**		**SDSCA**		**HbA1c**	
		**M ± SD**	**P value**	**M ± SD**	**P value**	**M ± SD**	**P value**	**M ± SD**	**P value**	**M ± SD**	**P value**
**Gender**											
Male	85(38.3)	6.9 ± 1.5	.631	4.3 ± 0.7	.066	4.5 ± 1.0	.051	3.5 ± 1.3	.405	6.6 ± 1.2	.979
Female	137(61.7)	7.0 ± 1.5		4.1 ± 0.7		4.3 ± 0.9		3.4 ± 1.2		6.6 ± 1.3	
**Marital status**											
Married	205(92.3)	6.9 ± 1.5	.954	4.2 ± 0.7	.453	4.4 ± 1.0	.614	3.4 ± 1.3	.875	6.6 ± 1.2	.453
Mateless	17(7.7)	7.0 ± 1.6		4.1 ± 0.7		4.5 ± 0.7		3.4 ± 1.3		6.8 ± 1.2	
**Age**											
Younger than 60	48(21.6)	6.7 ± 1.5	.509	4.1 ± 0.5	.466	4.4 ± 1.1	.897	3.4 ± 1.1	.610	6.5 ± 1.3	.925
60~	119(53.6)	7.0 ± 1.5		4.2 ± 0.7		4.4 ± 0.9		3.4 ± 1.2		6.6 ± 1.4	
Older than70	55(24.8)	7.0 ± 1.4		4.3 ± 0.8		4.3 ± 1.0		3.6 ± 1.5		6.6 ± 0.9	
**Education**											
Illiteracy or elementary school	21(9.5)	7.3 ± 1.6	.434	4.2 ± 0.6	.217	4.4 ± 0.6	.068	3.4 ± 1.3	.243	6.7 ± 0.9	.308
Junior high school	85(38.3)	6.8 ± 1.4		4.1 ± 0.6		4.6 ± 0.8		3.2 ± 1.2		6.4 ± 1.1	
Senior high school	64(28.8)	6.8 ± 1.6		4.1 ± 0.8		4.2 ± 1.1		3.6 ± 1.3		6.7 ± 1.4	
Technical school or college	52(23.4)	7.1 ± 1.5		4.4 ± 0.9		4.5 ± 1.0		3.5 ± 1.3		6.7 ± 1.5	
**Family per capita month income**											
Less than 2000 RMB	102(45.9)	6.9 ± 1.6	.464	4.2 ± 0.7	.652	4.5 ± 0.8	.314	3.5 ± 1.3	0.435	6.7 ± 1.3	.066
2000 or more RMB	120(54.1)	7.0 ± 1.3		4.2 ± 0.7		4.3 ± 1.1		3.4 ± 1.2		6.4 ± 1.1	

### Diabetes characteristics of participants

On average, duration of diabetes was 8.3 years (SD = 6.4, median = 7.0, range: 1–42). Eighty-six participants had a family history of diabetes (38.7%). Most reported they didn’t have clinical symptoms (56.8%), while 69.8% had complications, such as hypertension, coronary disease, stroke or kidney disease, et al. And most of them were centrally obese (70.7%). HbA1c values of participants with central obesity were significantly higher than those of normal participants. The longer the duration of diabetes, the higher the HbA1c value (see Table 2).

**Table 2:** Diabetes characteristics of participants

**Characteristic**		**N(%)**	**Self-efficacy**		**Social support**		**PPC**		**SDSCA**		**HbA1c**	
			**M ± SD**	**P value**	**M ± SD**	**P value**	**M ± SD**	**P value**	**M ± SD**	**P value**	**M ± SD**	**P value**
**Family history of diabetes**												
Yes		86(38.7)	6.9 ± 1.5	.990	4.2 ± 0.7	.561	4.3 ± 1.0	.266	3.3 ± 1.3	.331	6.5 ± 1.3	.456
No		136(61.3)	6.9 ± 1.4		4.2 ± 0.7		4.4 ± 0.9		3.5 ± 1.3		6.6 ± 1.2	
**Duration of diabetes(years)**												
~4		64(28.8)	6.7 ± 1.4	.382	4.2 ± 0.6	.474	4.1 ± 1.2	.018	3.3 ± 1.3	.350	6.0 ± 0.7	<.001
5~		72(32.4)	7.0 ± 1.6		4.3 ± 0.8		4.6 ± 0.8		3.6 ± 1.4		6.7 ± 1.5	
10~		56(25.2)	7.1 ± 1.2		4.2 ± 0.7		4.4 ± 0.8		3.5 ± 1.2		6.7 ± 1.1	
15~		30(13.5)	6.8 ± 1.7		4.0 ± 0.6		4.3 ± 0.9		3.1 ± 1.2		7.2 ± 1.3	
**Clinical symptoms**	Yes	96(43.2)	6.8 ± 1.6	.244	4.2 ± 0.7	.612	4.5 ± 0.9	.333	3.4 ± 1.3	.803	6.8 ± 1.4	.069
	No	126(56.8)	7.0 ± 1.4		4.2 ± 0.7		4.1 ± 1.1		3.4 ± 1.2		6.4 ± 1.1	
**Complications**	Yes	155(69.8)	6.9 ± 1.5	.509	4.3 ± 0.7	.042	4.5 ± 0.9	.012	3.4 ± 1.3	.686	6.6 ± 1.2	.885
	No	67(30.2)	7.0 ± 1.4		4.0 ± 0.8		4.1 ± 1.1		3.5 ± 1.3		6.6 ± 1.2	
BMI*	<24	91(41.0)	7.1 ± 1.5	.285	4.1 ± 0.7	.062	4.3 ± 0.9	.343	3.4 ± 1.2	.485	6.6 ± 1.3	.880
	≥24	131(59.0)	6.9 ± 1.4		4.2 ± 0.8		4.4 ± 1.0		3.5 ± 1.4		6.6 ± 1.2	
**Central obesity#**	No	65(29.3)	6.7 ± 1.6	.137	4.1 ± 0.7	.408	4.4 ± 0.9	.990	3.5 ± 1.2	.766	6.2 ± 0.8	.002
	Yes	157(70.7)	7.0 ± 1.4		4.2 ± 0.7		4.4 ± 1.0		3.4 ± 1.3		6.7 ± 1.3	

### Effects of self-efficacy, social support and PPC on SDSCA and HbA1c

**Figure 2:**
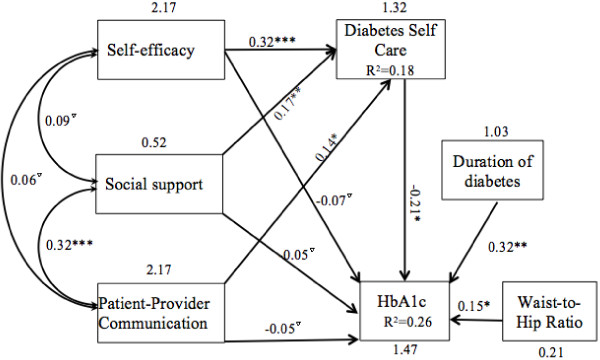
**Estimated model of the relationship of self-efficacy, social support, patient-provider communication, and their effect on diabetes self-care and glycemic control.** Note: Coefficients are standardized path coefficients. Overall model fit, *χ*^2^ (12, N = 222) = 1.71, p = 0.23, CFI = 0.96, SRMR = 0.04, RMSEA = 0.05. For tests of significance of individual paths, ^▽^p > 0.05,*p < .05, **p < .01 and ***p < .001.

Because central obesity and duration of diabetes were associated with HbA1c, so which were included in the model. The estimated MVPA with parameters and statistical significance of individual paths is shown in Figure 2. The estimated model demonstrated good model fit, *χ*^2^ (12, N = 222) =5.73, p = 0.23, CFI = 0.96, SRMR = 0.04, RMSEA = 0.05. As indicated in Figure 2, there were significant negative direct effects from SDSCA (β = −0.21, p = .007) to HbA1c, and positive direct effects from duration of diabetes(β = 0.32, p = .005) and waist-to-hip ratio(β = 0.15, p = .009) to HbA1c. The model explained 26% of the variability in HbA1c. There were significant positive direct paths from self-efficacy (β = 0.32, p < .001), social support (β = 0.17, p = .009) and PPC (β = 0.14, p = .029) to SDSCA, explaining 19% of the variability in the diabetes self-care behaviors score. Additionally, PPC was positively associated with social support(γ = 0.32, p < .001). Although Self-efficacy, social support and PPC had no direct effect on HbA1c, all of them had an indirect effect on HbA1c (β =–0.06, β =–0.04, β =–0.03 respectively) through SDSCA.

## Discussion

The main goal of diabetes management is glycemic control. This study indicated that only 52.7% of participants achieved the glycemic control target of HbA1c < 6.5% as recommended by the Chinese Diabetes Society [4]. Self-care is crucial in diabetes management because self-care behaviors can influence glycemic control. Although many studies have analyzed the effects of self-care behaviors, self-efficacy, social support and PPC on glycemic control, the relationships among these variables are still not clear. In this study, we used structural equation models to clarify these relationships and their effects on glycemic control. The findings demonstrated that more self-care behaviors were directly associated with better glycemic control after controlling for duration of diabetes and waist-to-hip ratio, which are factors associated with poor glycemic control [30-32]. These findings confirm previous studies’ conclusions that self-care behaviors benefit glycemic control [5-7,33].

Some prior studies indicated that self-efficacy [12,14,34], social support, and PPC [19] predicted better glycemic control. The current study suggests these constructs do not have direct effects on glycemic control; rather, they influence glycemic control indirectly through self-care behaviors. Previous studies also reported social support as a significant predictor of self-care behaviors, which, in turn, affect glycemic control [35,36]. Similarly, past studies have shown that greater self-efficacy lead to greater self-management [10], in turn leading to better glycemic control [13].

Although self-efficacy, social support and PPC have direct effects on self-care behaviors, only social support and PPC were correlated with each other. This suggests that physicians may be a main source of participants’ social support. Because more than 40% of participants identified their physician as the person who provides the greatest assistance in managing their diabetes [16]. Univariate analysis of the current study indicated that diabetic patients with complications had better PPC than those without complications. Chinese patients usually rely on the physician’s suggestions for disease treatment [10]. Furthermore diabetic patients with complications need to visit physician more frequently than those without complications, which may improve patients’ knowledge, and help physician understanding them comprehensively resulting to fluent patient-physician communication.

### Limitations

There are limitations to this study that should be acknowledged. First, our results speak most clearly to the population under study, but most were older and had complications. Therefore, this study should be replicated in different patient groups. Secondly, we were unable to explore the role of moderators (e.g., medication type, literacy level, gender) in the evaluated models due to a restricted sample size.

In addition, the current study measured these constructs cross-sectionally, and thus can most appropriately speak to associations between constructs observed at a single point in time, not causality. Future research should be conducted to investigate the longitudinal effects of self-efficacy, social support and PPC on changes in diabetes self-care behaviors and glycemic control.

## Conclusions

Despite these limitations, this study is the first to our knowledge to explore the relationships of self-efficacy, social support and PPC, and their effects on self-care behaviors and glycemic control among Chinese patients with type 2 diabetes in a whole model. Specifically, having better PPC, having higher social support, and having higher self-efficacy was associated with performing diabetes self-care behaviors; and these behaviors were directly linked to glycemic control. So longitudinal studies are needed to explore the effect of self-efficacy, social support and PPC on changes in diabetes self-care behaviors and glycemic control.

## Abbreviations

HbA1c: Glycosylated hemoglobin; DSMS: Diabetes self-management support; PPC: Patient-provider communication; SDSCA: Summary of diabetes self-care activities.

## Competing interests

The authors declare that they have no competing interest.

## Authors’ contributions

JLG and HF conceived the study and JLW and YCZ collected the data. JLG, RH and MCK worked on the analysis and polished the language. All authors participated in drafting the article and final reviews. All authors read and approved the final manuscript.

## Pre-publication history

The pre-publication history for this paper can be accessed here:

http://www.biomedcentral.com/1471-2296/14/66/prepub
